# Creation of Work Integration Social Enterprises (WISEs) by Social Action Organizations: Proposal of a Model for Decision-Making

**DOI:** 10.1007/s11266-021-00447-2

**Published:** 2022-02-07

**Authors:** Francisco Pizarro Escribano, Francisco Javier Miranda González

**Affiliations:** 1 University of Extremadura, Badajoz, Spain; 2 FundecytPCTEX, Badajoz, Spain

**Keywords:** Social enterprise, Social entrepreneurship, Third sector, Work insertion social enterprise, Entrepreneurial process, Entrepreneurial NGOs

## Abstract

Successive crisis in Europe have contributed to rethink welfare state and the entrepreneurial role of Third Sector organizations in the provision of community services that progressively have created social enterprises. Its creation is the result of a decision-making process that is collective, not individual, and of a strategic nature, in which the organization's culture plays a relevant role. This work aims to describe and analyze the entrepreneurial process, and the key elements that determines the decision of creating a work insertion social enterprise by its promotor entity. As a result, this article proposes an explicative model of social enterprises creation and makes an empirical validation, using Delphi Method in Spanish work insertion social enterprises case.

## Resumen

Las sucesivas crisis en Europa han contribuido a una puesta en cuestión del estado de bienestar y, con ello, al rol emprendedor o productivo de las organizaciones del Tercer Sector en la provisión de servicios a la comunidad, en la medida en que éstas han creado empresas sociales progresivamente. Su creación es el resultado de un proceso de toma de decisión que es colectivo, no individual, y de naturaleza estratégica, en el que la cultura de la organización juega un rol relevante. Este trabajo trata de describir y analizar el proceso emprendedor y los elementos clave que determinan la decisión de crear una empresa de inserción social, por parte de su entidad promotora. Como resultado, este artículo propone un modelo explicativo de la creación de empresas sociales y realiza una validación empírica con las empresas de inserción españolas, utilizando el método Delphi.

## Introduction

After the crisis of the 1990s, with the birth of active labor market policies (ALMPs), the social action “Third Sector” was called upon to play an entrepreneurial role to compensate for the state and market failures that stem from the increasing rates of structural unemployment (Borzaga & Santuari, 2001).

Then, just when people, companies, institutions, and community organizations were starting to adapt to the new labor market rules after the 2008 crisis, the economic and social crisis accompanying the health crisis caused by COVID-19 posed a fresh new test to the employment-exclusion binomial. In this context, non-profit organizations were committed again to create social enterprises for the labor insertion of excluded people.

Thus, the main objective of this work is to identify the environmental, organizational, and economic factors that influence the decision to create a work insertion social enterprise (WISE) by its promoting entity. At the same time, it aims to contribute to the study of social entrepreneurship as an organizational process.

Social enterprises constitute a heterogeneous group, and they are specifically regulated by their social goals and their geographical locations. In order to have a homogenous subject of study, we will focus on the creation process of Spanish WISEs. One of the conditions that Spanish regulations require of a WISE is that it must be owned by a non-profit organization that acts as a promoter. In this way, we can be sure that all of the participants in this empirical study involve organizational social entrepreneurial processes and not individual.

Thus, the study of this entrepreneurial form brings us into the realm of social entrepreneurship, not so much as an individual activity with a broad motivation for the solution of a social problem, as presented in the dominant academic current (Dees, 1998; Drayton, 2002; Mair and Martí, 2006), but rather as an organizational and strategic process within a social action organization (Dorado & Ventresca, 2013; Kannampuzha & Hockerts, 2019). This organizational conception of social entrepreneurship is increasingly important in the academic literature, especially in continental Europe (Defourny et al., 2014).

Therefore, we believe that the case of Spain can be an interesting example to analyze the decision-making process of organizations when creating a social enterprise within the European stream as opposed to the Anglo-Saxon stream.

As a result, this article contributes to the literature on social entrepreneurship and the management of non-profits with a validated model for decision-making on the creation of social enterprises by social non-profit organizations and, specifically, on the creation of WISEs.

To do this, we have carried out a qualitative study, using the Delphi method, through surveys of experts who have participated in the decision to create almost 50% of the 250^1^ WISEs registered in Spain.

## Social Entrepreneurship as an Organizational Process

In the mainstream of social entrepreneurship, with a marked Shumpeterian character, it is understood as an eminently individual act, where the search for the innovative entrepreneur who tries to transform pre-existing structures for the generation of social value is key in the social qualification of the managerial outcome (Dees, 1998; Drayton, 2002).

However, the most recent literature identifies two key aspects salient to the objectives of the present research.

On the one hand, social entrepreneurship is understood, not as a mere economic act, but as a complex process of discovering an opportunity to generate social impact, which involves a series of phases leading up to the creation of the social enterprise (Guclu et al., 2002; Dorado and Haettich, 2004; Hockerts, 2006; Mair and Martí, 2006).

On the other hand, social entrepreneurship, especially in Europe, is understood as being not so much an individual process as the result of collective dynamics involving members of a particular community or group who share a common need or goal (Borzaga & Defourny, 2001). One could even speak of an evolutionary trend of the Third Sector itself toward a productive role in transforming the welfare state into a welfare society (Rodríguez Cabrero, 1994; Borzaga et al., 1996; Dart, 2004).

In this context, a new concept has fittingly been coined, organizational social entrepreneurship, defined by Kannampuzha and Hockerts (2019) as the activities of organizations created with the primary objective of generating social impact for their beneficiaries by participating in commercial activities while employing cooperative mechanisms of governance to defend the primacy of their beneficiaries.

More recently, Cordobés et al. (2020), based on interviews with 500 professionals from the Third Sector and reviewing the most recent literature, identified eight trends that determine a growing interest in the entrepreneurial activity of NGOs: collaboration for entrepreneurship and innovation, systemic changes post-crisis, digitization, transparency, gender and diversity, talent reskilling and leadership, new vision in uncertain contexts, and intrapreneurial policies.

The conceptual challenge in our case is uniting these two streams in the literature, to understand and describe the entrepreneurial process within a non-profit organization that decides to take on a role, not only innovative and demanding, but also productive, and launch a market-oriented business activity to achieve its ends.

We begin by considering the social entrepreneurial process as the result of the continual interaction of its promoters with their context (Mair and Martí, 2006), with the environment, from a more strategic perspective, or with its ecosystem, as analyzed in the most recent literature, precisely as a synthesis between environment and context, that is, agents, relationships, norms, and the prevailing circumstances (European Commission, 2020).

This iterative process, between the environment and the organization, allows for assessing exogenous opportunities and for taking advantage of the endogenous resources, capacities (Guclu et al., 2002) and entrepreneurial culture of the organization (Stevenson & y Gumpert, 1985).

Perrini and Vurro (2006), based on an extensive review of the literature, explain the social entrepreneurial process in three phases: (a) the definition of the opportunity; (b) the design, launch, and functioning of the organization; and (c) the strategy for financing the venture and obtaining the necessary resources. However, from our point of view, these three elements do not have to be consecutive, but rather are three transversal axes that must evolve in the conceptual, entrepreneurial, and organizational phases of corporate entrepreneurship (Burgelman, 1983).

At all times, it is necessary to pay simultaneous attention to the social and the business aspects, since it reflects the hybrid nature of the social business, and to recognize the social impact that can be generated while also making sure of its sustainability over time.

The planners must then map out the social assets in the community (Kretzmann & McKnight, 1996), understood as potential generators of social capital, the fruit of the relationships that the company can establish from its inception. Those assets include those of the promoting entity for carrying out the project.

While the problem to be solved constitutes a priority for the social entrepreneur, the decision to start an enterprise must be based on the possibility of establishing the organizational formula and of launching an economic activity that will successfully mobilize the necessary resources (Austin et al., 2006), while being able to contribute to both the primary objectives. That is, the social goals of job placement, and the secondary objectives, those involving the production of goods or services of general interest, in the most effective way possible (Guclu et al., 2002) through a proposal of mixed value, social, and economic (Emerson, 2003), sustainable and sustained over time.

The business model describes the bases on which a company creates, provides, and captures value. Since the popularization of the canvas tool for the design of business models by Osterwalder and Pigneur (2010), there have been many proposals for redesign of the tool for social companies (Petrini et al., 2016; Sparviero, 2019), and not only for these, but to analyze the threefold set of results—financial, social, and environmental (Elkington, 1998)—that an idea for a lucrative business can achieve, the use of the triple-layer business model canvas has become widespread (Joyce & Paquin, 2016).

Thus, in the design of the business model of a social enterprise,^2^ the elements of commercial activity should be combined with others related to the generation of social value^3^ for the clientele; the beneficiaries and other stakeholder groups must be considered. Consequently, in the second place, to the commercial activity must be added the social process that takes place within the company. Third, in addition to calculating the economic benefit equation, it is necessary to express how and where the positive result will be reinvested, and the indicators by which the expected result and social impact will be measured. Fourth, and perhaps previously, some authors (Sparviero, 2019) introduce more strategic elements of the social enterprise, such as the mission, values, objectives, and system of governance.

Finally, it is necessary to consider key aspects of the organizational formula of the company being created. The promoting organization must consider the constituent elements of the nature of the social enterprise^4^ and those of the WISE.^5^ As noted, the social and the business aspects of the undertaking must be designed in a coordinated and coherent manner. The productive structure in the WISE is conditioned by the necessary combination of a mix of human resources including integration and placement support staff, production specialists, and the beneficiaries themselves that will affect its organization and its productivity to meet the social objective.

For this, it must be flexible and able to integrate diverse interests and adapt to the environment, using the knowledge that surrounds it for specific productive objectives, as indicated by the evolutionary theory of firms (Nelson & Winter, 1982). Therefore, the objective in the creation phase of the company is to determine the organizational formula necessary for the start-up, and not so much to design it as if it were in a later stage of maturity and operating at full capacity.

Specifically, the organizational formula of the social enterprise must also resolve internal agency relationships, coordinating the different stakeholders whose motivations and interests may be in conflict. So that the hierarchy, control, and extrinsic economic incentives typical of vertical organizations lose their importance in favour of coordination, participation, and the intrinsic nonpecuniary motivations associated with more horizontal organizations (Borzaga & Tortia, 2006). The organization designed with these criteria contributes to granting the legitimacy that fosters relationships of trust and commitment among all the actors involved in the processes, both business and social service, reducing transaction and production costs.

**Fig. 1:**
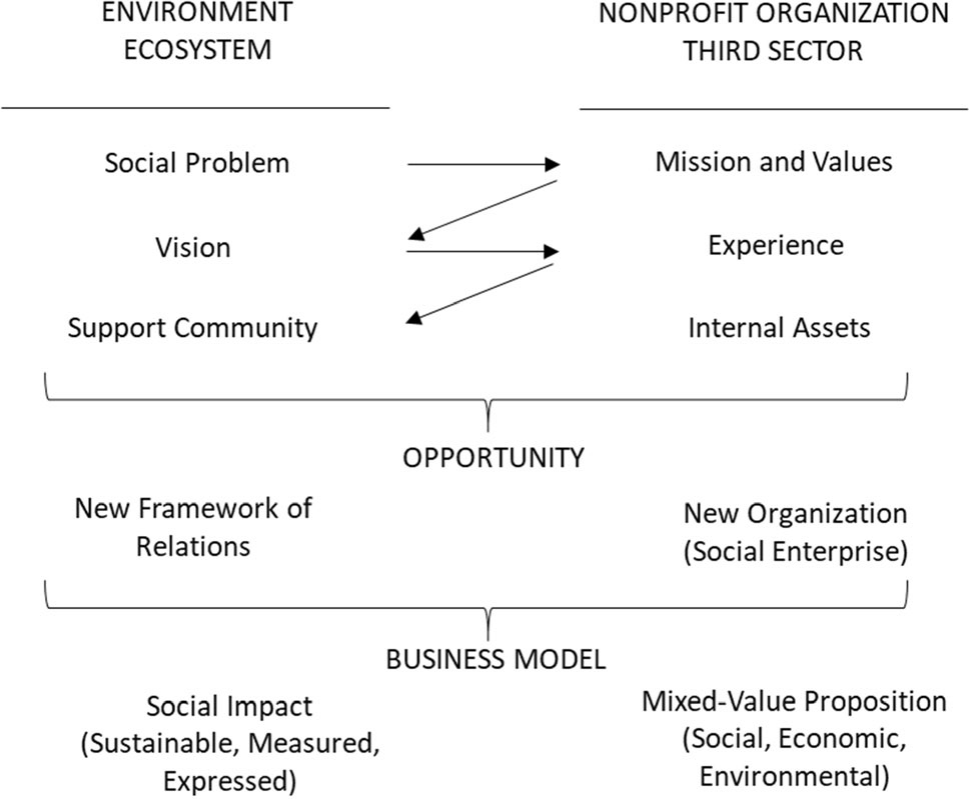
Decision by a Third Sector organization to create a social enterprise. Own elaboration

Thus, the decision by a Third Sector non-profit organization to create a social enterprise, once the opportunity has been identified in business terms, entails reviewing its coherence with its fundamental social objectives, calculating its likely social and environmental results and impact, identifying the availability of endogenous resources for its implementation, and determining the organizational model and the relationship framework that will make it possible to manage the complex nature of the social enterprise (see Fig. 1).

In the case of social enterprises for labor insertion, the opportunity, in social terms, emanates from limitations of the market (Drucker, 1985) in the employability process, especially for people in a situation or risk of social exclusion. This problem is bigger after a crisis and the resulting structural changes, where mobility and new competences are demanded of workers while technology has modified the salary-productivity ratio. In these contexts, employment policies prove ineffective (Laville, 1998).

For this reason, the social action Third Sector in Europe promoted and managed structures, services, and methods of socio-labor integration of people who were either excluded or at risk of exclusion, drawing from the social economy a set of cooperative and person-centered managerial principles and leading to what in the 1990s was considered a radical methodological innovation (Laville, 1998).

It could be said that three decades later, the mere creation of a social enterprise to facilitate job training and experience prior to transition into the labor market (i.e., a WISE) cannot be classified as a social innovation.

From the point of view of social entrepreneurship, one could even regret that the progressive regulation of this type of company might limit the innovative responses of this phenomenon. After twenty years of work insertion social enterprises, and near 200 created, Spanish law 44/2007 came as the culmination of several ongoing academic debates about the nature of the WISE (López-Aranguren, 1999; Retolaza, 2010; Ruiz Roqueñi et al., 2007).

In Spain, if a promoting entity wants the created company to be classified and registered as a WISE, thereby obtaining the corresponding public, labor, fiscal, and financial benefits, it must meet the following criteria:

- It must be constituted with a commercial or cooperative legal form.
- It must carry out a market economic activity, while at the same time its corporate purpose must be labor insertion.
- At least 80% of its net proceeds must be reinvested in productive activity or in the process of social integration.
- From its fourth year of operation, at least 50% of its total workforce must be beneficiaries of the social integration program.
- It must be a transitional employer: its beneficiaries must have a contract for a maximum of three years.
- The company must provide its beneficiaries with a personalized itinerary for the duration of their work contract.

But the most relevant criterion for this research is that the company must be promoted by, and run with the active participation of, a social action organization dedicated to the employment and social integration of people who are either experiencing exclusion or at risk thereof.

Nowadays, 250 WISEs are registered in Spain under this legislation, generating more than 4,000 social jobs, with a 68% rate of insertion into the labor market, incomes of 143 million euros, and with only 20% of their budget coming from public aid (FAEDEI, 2019).

## Theoretical Model for the Decision to Create a WISE

Based on the theoretical framework analyzed above, a synthesis can be constructed representing the decision process that occurs within a social action non-profit organization that considers the creation of a WISE as a continuation of its agenda of social integration.

Thus, the organization must analyze in advance its mission and vision, its entrepreneurial culture, and its internal assets (relationships, reputation, knowledge, team, finances). Ideally, the decision should not be a mere managerial or administrative process, but rather an innovative and entrepreneurial process that gives rise not to just another WISE capable of obtaining the requisite administrative credentials, but rather, to a genuinely new social enterprise.

For this, a process of organizational, or corporate, social entrepreneurship in three phases is proposed:

- *The conceptual phase*, which consists of the consolidation of the perception of the desirability of starting a company, considering the organization, the circumstances, and the beneficiaries’ needs.
- The *entrepreneurial phase*, in which the business model and the viability of the WISE to be created are analyzed.
- The *organizational phase*, in which the decision to start the company is finalized as a set of operational, organizational, financial, and legal-administrative decisions, considering the criteria identified in the previous phases.

Thus, throughout the process, variables involving the environment and the promoting entity must be analyzed along three transversal axes:

- *Environmental. The determination of the opportunity*, appropriate to the circumstances and with the potential to generate economic and social results that will allow for a sustained social impact over time.
- An *organizational design* that not only allows the commercial activity to be carried out with a quality standard, but also incorporates the requisite processes of social and labor integration and involves the stakeholder groups and legitimizes the company as a public service enterprise.
- *Design of the strategy for attracting resources*, according to the hybrid model of a social enterprise, combining commercial activity with the philanthropic contributions from its support community and the grants from the state as a structure that contributes to active employment policies.

In addition, for the focus of the present study, promoters must consider the requirements that Spanish law and various regional regulations impose for administrative recognition as a WISE, very similar to others in Europe.

We can summarize these requirements, roughly, in four groups: *the social purpose of WISEs,* the *transitory model* to the regular labor market, the *non-profit nature* that requires that they must be promoted by and/or involve the participation of a non-profit organization, and *administrative registration,* which implies accountability obligations, preparation of activity reports and social balance sheets.

**Fig. 2:**
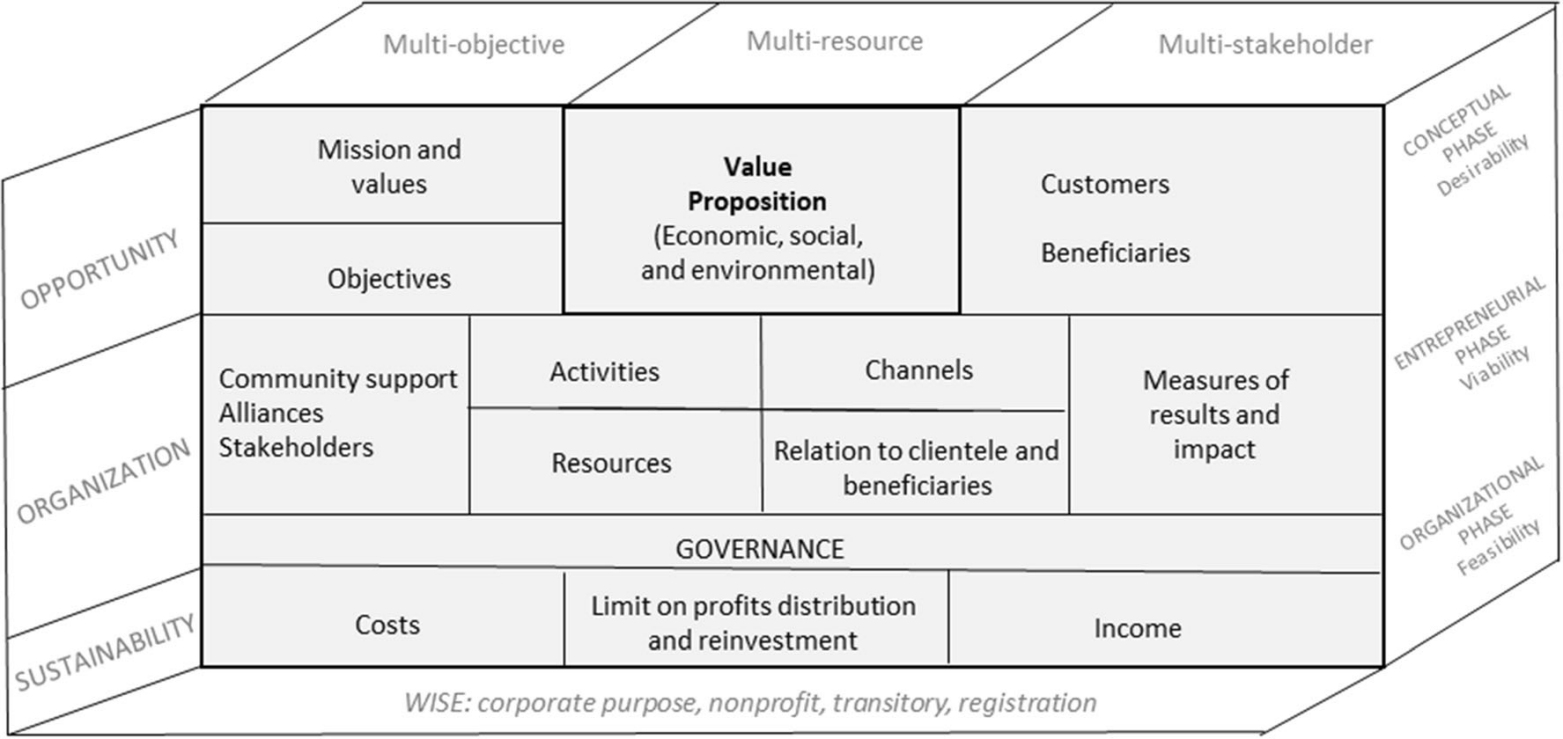
Model for the decision to create a WISE. Own elaboration based on Defourny and Nyssens (2010), Sparviero (2019), Burgelman (1983), Mair and Noboa (2006), and Perrini and Vurro (2006)

The model proposed as a synthesis of the theoretical elements reviewed above is summarized in Fig. 2.

Therefore, the main contribution of our article will be to describe in detail the different phases that make up the decision process to be followed when creating a social enterprise, as well as to analyze the different criteria that can be used in this decision-making process.

## Methodology of the Empirical Study

Once the theoretical model has been defined, it can be empirically validated. For this, we have opted for a qualitative methodological approach, namely the Delphi method (Dalkey & Helmer, 1963). Because the universe of study is small, homogenous, and recent, we can count on the participation of experts with very relevant opinions on the object of the research, and the methodology allows us to obtain a high level of consensus between the answers, prioritizing the opinion of the group over the individual ones, as well as a high level of validity with relatively few answers for a quantitative approach, but high for the qualitative methodology.

This method has been used previously to study WISEs as social entrepreneurship (Melián et al., 2011), though the objective of that research was to construct an entrepreneurial profile of the person who promotes them. For the present study, we will explore their creation as an organizational process within the promoting entity, seeking to identify criteria for joint and institutional decision-making.

The Delphi methodology seeks consensus among a group of experts on the subject under study through an interactive process in which, after they have answered a questionnaire individually, a new questionnaire is then issued, where participants are informed of the answers obtained from the first questionnaire and allowed to modify their initial responses to obtain a consensus.

The properties of the Delphi methodology will allow us to validate the stages of the proposed decision-making model, as well as to identify the criteria that the experts consider most important when systematizing this process and which, therefore, are expected to be key in the process of creating new social enterprises in the coming years.

To accomplish this, the first step was to develop a questionnaire based on the theoretical analysis of both the figure of the WISE as a social enterprise and the process of social entrepreneurship in tandem with corporate entrepreneurship. As a result, the questionnaire is structured in three blocks of criteria to be considered in the decision to create or not to create a WISE, each with two perspectives, one internal to the organization, and the other external to the social and economic context in which the decision is made (Bird, 1988; Krueger, 1993; Mair and Noboa, 2006; Coduras et al., 2011; Zahra et al., 2009):

- A first block refers to desirability criteria—that is, if the circumstances of the social environment of the organization call for the creation of a company for the fulfilment of its purposes (6 items) and if the necessary starting conditions to consider the possibility of creating it are met (8 items) (1, totally disagree, to 7, totally agree) (López-Aranguren, 1999; Marcuello et al., 2008).
- The second block refers to the viability criteria. This involves reviewing the resources and capabilities of the organization that could address specific business opportunities or determine the characteristics of the economic activities that could be considered (1, not important at all, to 7, very important) (Chaves & Sajardo, 1999; Retolaza, 2010).
- The third block refers to the feasibility criteria. Here, the respondents were presented with specific options for the creation of the company, derived from the institutional context or the specific legal framework of WISEs and asked to distinguish those factors that facilitate the creation process from those that make it difficult (1, makes its creation easier, to 7, makes its creation more difficult) (Campos et al., 2014; Alves, 2012).

Once the questionnaire had been drawn up, based on the theoretical model, the panel of experts had to be selected. Here, the objective was not so much to interview managers or promoters of social enterprises, but rather, senior staff of social action entities who have participated in the decision-making process of creating, or not creating, a WISE as a new structure in their agenda for the fulfilment of their objectives of socio-labor integration.

The questionnaire was sent by email to 162 professionals who, working for social entities, might have participated in the decision to create a WISE, with a letter explaining the object of the study and the method being used. This resulted in the participation of 48 experts, of whom 6 had not participated in the process of creating a company, while the remaining 42 had.

It needs to be noted that the evolution of the creation process of this type of company has given rise to the creation of networks of WISEs started by the same organization, so these 48 experts have been involved in approximately 50% of the WISEs registered throughout the national territory.

The experts were surveyed for the first time between the months of March and May 2020, and for the second time in the month of June 2020.

The data from the completed questionnaires were tabulated, and descriptive statistics were calculated for each of the data, calculating measures of centralization, position, and dispersion, detailing the means, standard deviations, and medians (see “Appendix”).

Already in the first round, 81.48% of the items reached a consensus higher than 60%, especially in blocks 1 and 2 of questions, with a standard deviation less than 2, so in the second round, an attempt was made to reach an even higher consensus, especially in block 3.

Consensus on each item was determined by the percentage of votes within a prescribed range (Hsu & Sandford, 2007). In this study, it was considered that a consensus was reached when the scores given by the participants did not deviate from the median by more than the limit of the standard deviation, in each of the items of the questionnaire.

**Fig. 3:**
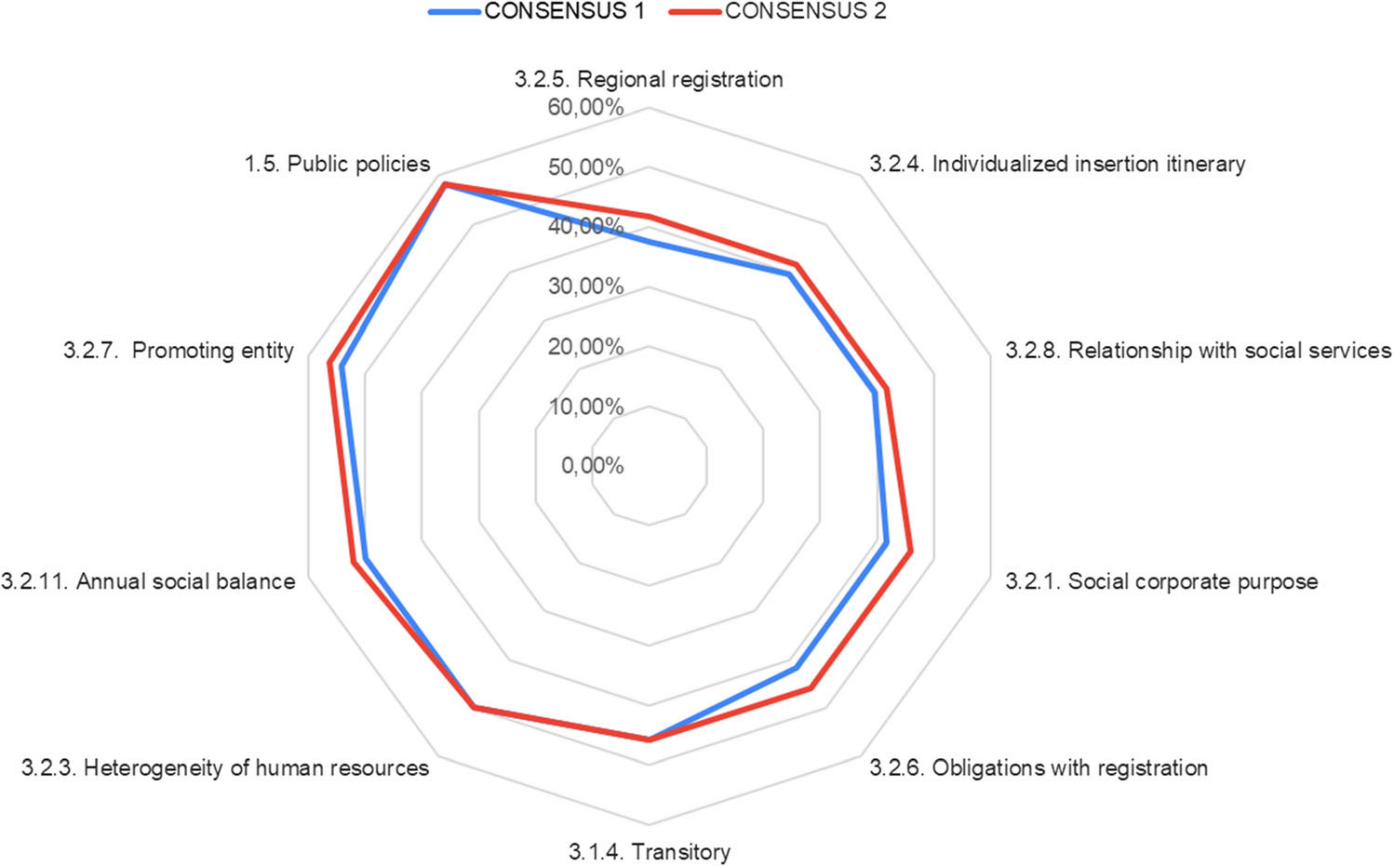
Items with the lowest level of consensus

Thus, the statements with the least consensus, and their modification in the second round, occurring mostly in block 3, in which it was deliberated whether the conditions of the social enterprise facilitate or hinder its implementation, are shown in Fig. 3.

These elements correspond with those most debated in the literature on the nature of WISEs, so that the perception about them depends on whether they are perceived as safeguards of the social nature of this type of structure, and therefore, as barriers to entry for initiatives not promoted by the Third Sector, or if, on the contrary, from a more entrepreneurial perspective, it is considered that they may hinder their viability, and therefore, make it difficult for more of these companies to emerge. In other words, a constraining factor can be perceived as something positive or negative depending on the expert’s point of view. In fact, these elements will be critical in future research surveying social organizations that perform labor integration services but have not yet created WISEs.

**Fig. 4:**
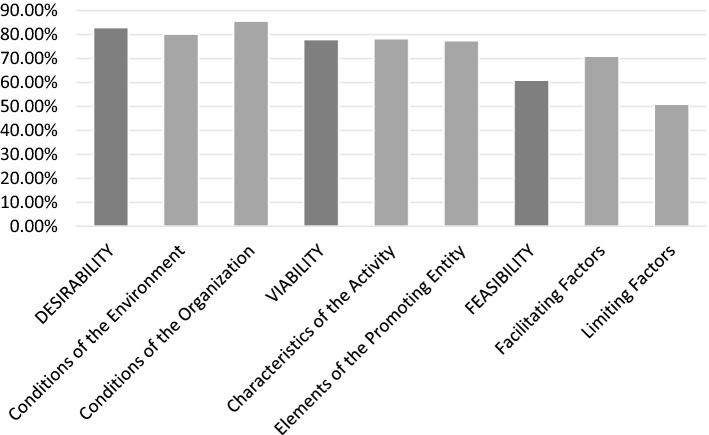
Level of consensus by blocks after the second round

In any case, as noted, most of the items presented high levels of consensus after the participating experts responded to the second round of questionnaires, with all blocks of questions showing consensus more than 50%. If we exclude the limiting factors of creation in the third block, the rest exceed 70% consensus (see Fig. 4).

## Results of the Empirical Work

**Fig. 5:**
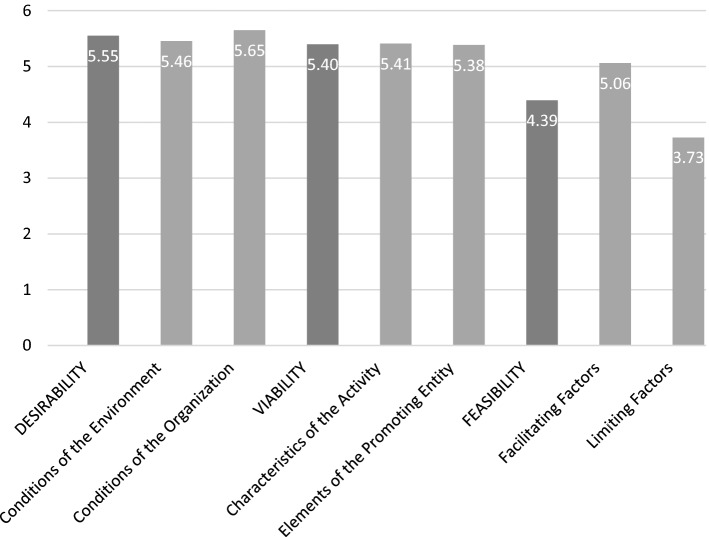
Process and criteria for the decision to create a WISE by its promoting entity

The assessment of the different criteria proposed for making the decision to create a WISE, on the part of its promoting entity, have been valued above the average (5 out of 7) by the experts consulted, as can be seen in graph 1. We must consider that in the last block, on limiting criteria, the evaluation is inverse (1 = makes its creation easier, 7 = makes its creation more difficult); therefore, the experts validate as adequate the specific requirements for the accreditation of the WISEs established in the regulation, which we have theoretically justified as assurances of the social enterprise nature of WISEs (see Fig. 5).

Within the homogeneity of the responses, considering the phases of the process, they give greater importance to the first phase of desirability (5.55 out of 7), in which the preconditions for the creation of the company are evaluated; and within them, those related to the organization itself (5.65 out of 7). The same happens in the viability phase (5.40 out of 7), second in importance, where more importance is given to the promoting entity’s resources (5.41) than to the characteristics of the economic activity (5.38), although with very little difference.

About the third phase, feasibility, where it is proposed to evaluate the importance of the requirements of the social enterprise and, specifically, those specific to WISEs that facilitate or hinder the creation of a business activity by a social action organization, it is worth highlighting their validation and practical neutrality (4.39 out of 7) when making the decision.

This is a relevant contribution, since, as we were able to analyze, the social nature of this type of company increases its complexity of organization and management. Notwithstanding, we found in contrasting interviews carried out that the very fact that we were analyzing the decision of launching a business venture within a non-profit organization, rather than individual action, caused the experts interviewed to assume the restrictions established for this type of company as positive and normal for ensuring its social nature.

If we analyze in detail the criteria proposed for each phase of the process, we will be able to validate the proposed model and identify priorities. To do this, we consider valid those criteria that have been evaluated at above 5 out of 7 and that do not present a dispersion in the responses greater than 2 (standard deviation < 1.4; variance < 2).

As we can observe in Table 2 (see “Appendix”), in the first phase, where the individuals who must make the decision evaluate the prior conditions and accordingly form their perceptions of the desirability of launching a business enterprise, the experts minimize the support of public policies (4.31) and the support of the community (4.04), giving greater importance to the context variables.

It should be noted that the survey was carried out during the confinement period caused by the COVID 19 pandemic, during which time an aggravated social crisis to follow the health crisis was anticipated. At the same time, the consequences of the 2008 financial crisis, which has transformed labor relations (6.33) and the skills required of job seekers (6.19), are still being felt in the labor market.

These deficits in the labor market, together with the tendency of the Third Sector to assume productive roles and its evolution toward social enterprise (6.10) and also with successful experiences that validate the usefulness of carrying out productive activities as a means of achieving job placement for people facing actual or potential labor exclusion (5.75), determine the conditions of the environment that lead social action entities to consider the possibility of creating companies as something desirable for the fulfilment of their ends.

**Fig. 6:**
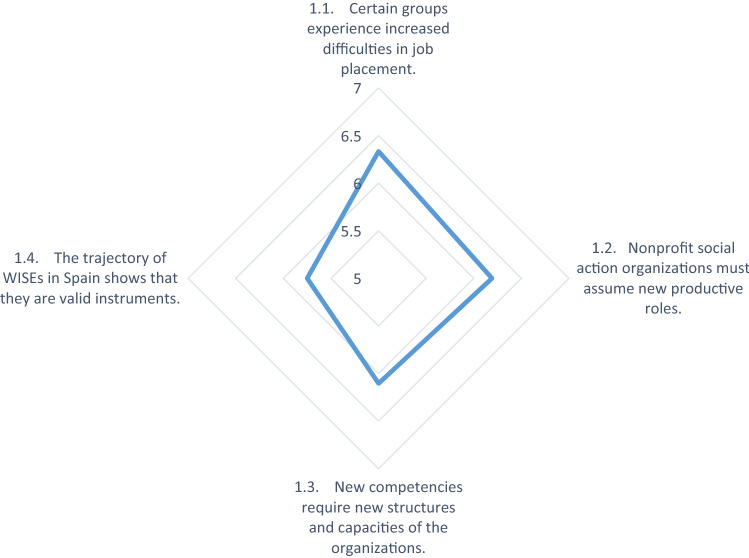
Conditioning factors of the environment

Figure 6 collates the criteria validated by the experts as conditioning factors of the environment that causes the managers of the promoting entities to feel either the desire or the need to create a WISE.

As for the conditioning factors of the organization itself, better valued as a whole than those of the context, they are key to the process as an organizational social enterprise. Thus, the process must rely, above all, on the commitment of the governing bodies (6.02), once it has been verified that the creation of a WISE is consistent with the organization’s strategy (5.83), in terms of both the entrepreneurial culture of the organization (5.79) and its fit into the job placement itinerary (5.79), especially in terms of adaptation to the profile of its beneficiaries (5.67). The entity’s ability to assume economic and financial risks seems a necessary condition to undertake the entrepreneurship process, but not as a priority (5.40).

**Fig. 7:**
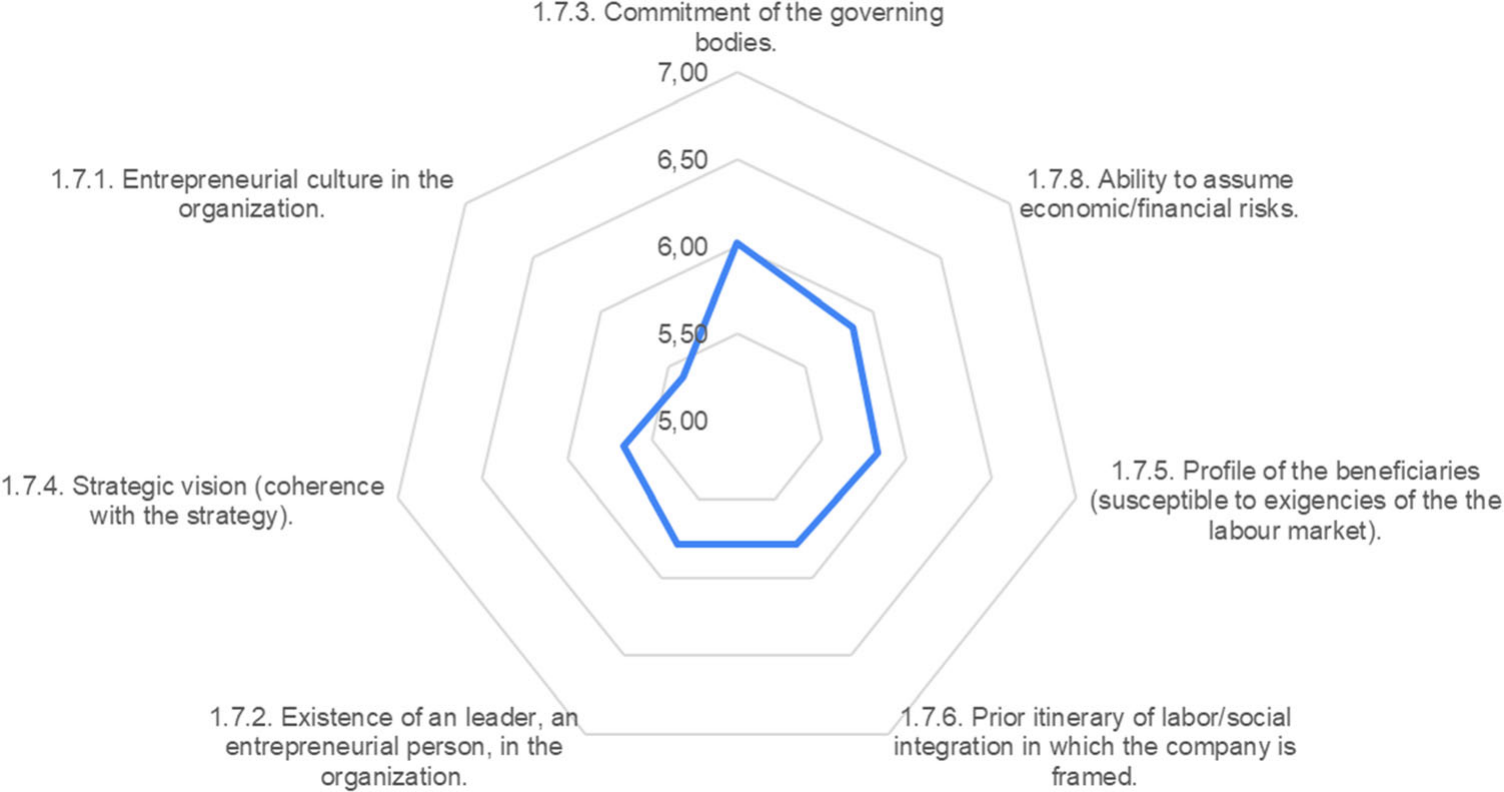
Conditioning factors of the organization

The reference of success stories as a condition for the desirability of launching a business by an organization does not have sufficient consensus among experts, and in any case, it seems a neutral factor (4.85), at least as it refers to cases specific to the environment, because as expressed in the context variables, the joint trajectory of the WISEs is already sufficient for understanding that they are a useful instrument (Fig. 7).

Regarding the second phase, the experts were asked to consider those criteria that may determine the opportunity to undertake an economic activity with a perception of the future viability of the business project. Once again, a distinction is made between those that depend on the organization’s resources and capacities and those that depend on the socioeconomic environment and possible economic activity (see “Appendix 2”).

Most of the proposed criteria obtain a favourable evaluation, higher than 5 on a scale of 1 to 7, and very homogenous, without one standing out too much over another. The proposed model is thus validated.

Before contemplating undertaking an economic activity or choosing the most appropriate one for the creation of a WISE, the promoting entity must ensure that it has the trained technical personnel necessary to start and manage it (5.58) and be aware of what competencies the beneficiaries may have acquired in occupational training workshops carried out previously (5.5). These capacities of the promoting entity can, however, be complemented with external methodological support (5.27), usually from public or civic institutions of the Third Sector that render reinforcement.

Likewise, the promoting entity must realistically assess its ability to assume the necessary initial investment, considering the limitation that the social enterprise will have in the distribution of profits (5.54) and making sure that there is an adequate network of relationships with public and private agents in the environment that will allow the multi-resource model of this type of company (5.48).

Thus, the first step in the phase of opportunity analysis and estimation of the future viability of the company is to make an inventory of the resources and capacities of the promoting entity, which will determine the human capital, financial capital, and relational capital that can be counted on.

**Fig. 8:**
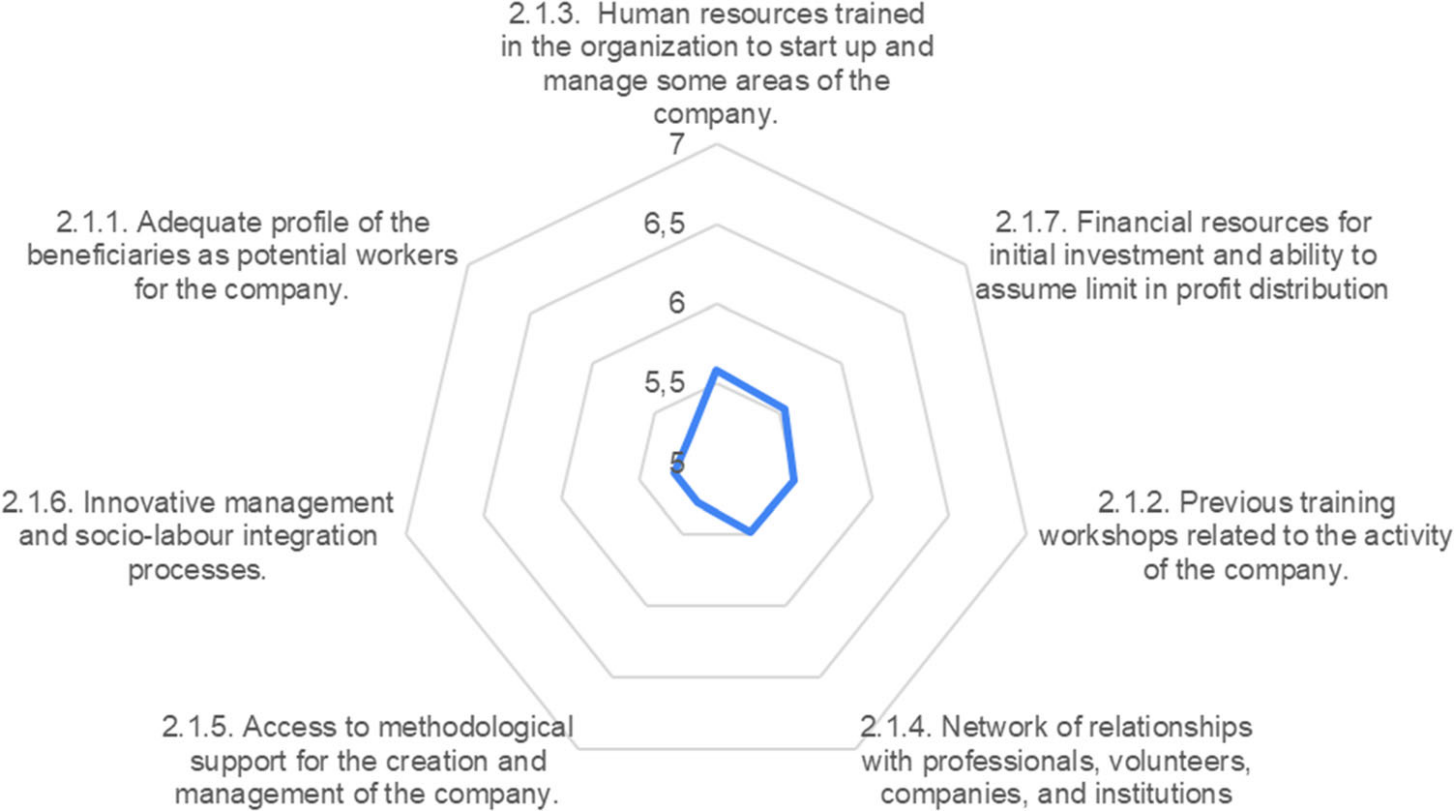
Characteristics of the organization

It must not be forgotten that the purpose of the WISE is to improve the employability of the organization’s beneficiaries, and that a new methodological structure will be made out of the previous insertion itinerary. Consequently, the methodological innovations previously developed by the organization (5.27), and the prior skill level achieved by its beneficiaries (5.23) are criteria to be evaluated in this process, since the social enterprise is not an end in itself, but an instrument to improve the performance of the organization’s principal function (Fig. 8).

After analyzing the organization, it is time to define the selection criteria for the business activity to be carried out, with a double priority of the social and business considerations. On the one hand, there are the criteria consistent with the social purpose, and on the other, those that determine its economic and financial viability, though the latter must also be considered in relation to the social assets of the promoting entity and its environment.

The experts put a premium on the criteria of demand, both in the free market (5.98) and in protected public purchase share (5.98). They consider it vital that the goods or services being offered respond to a need expressed by the market or by public administrations, usually municipalities, and even that there be an assured level of purchase if it is carried out. In fact, the ideal is to reduce the amount of commercial action necessary (5.23), even if this means entering value chains with stable demand. What matters is that the margins obtained allow the sustainability and reinvestment capacity necessary to maintain competitiveness (5.70).

The economic criterion is necessary, but not sufficient. Next, its suitability for its social purposes must be verified. Most fundamentally, it must be fitted to the capacities of the beneficiaries (5.91), and if possible, it should also generate social or environmental value for the clientele and the community (5.88). Along these lines, it would be suitable that the economic activity will be consistent with the training actions previously carried out by the organization in its insertion itinerary (5.19) and its ability to generate the greatest possible job placement (5.08).

**Fig. 9:**
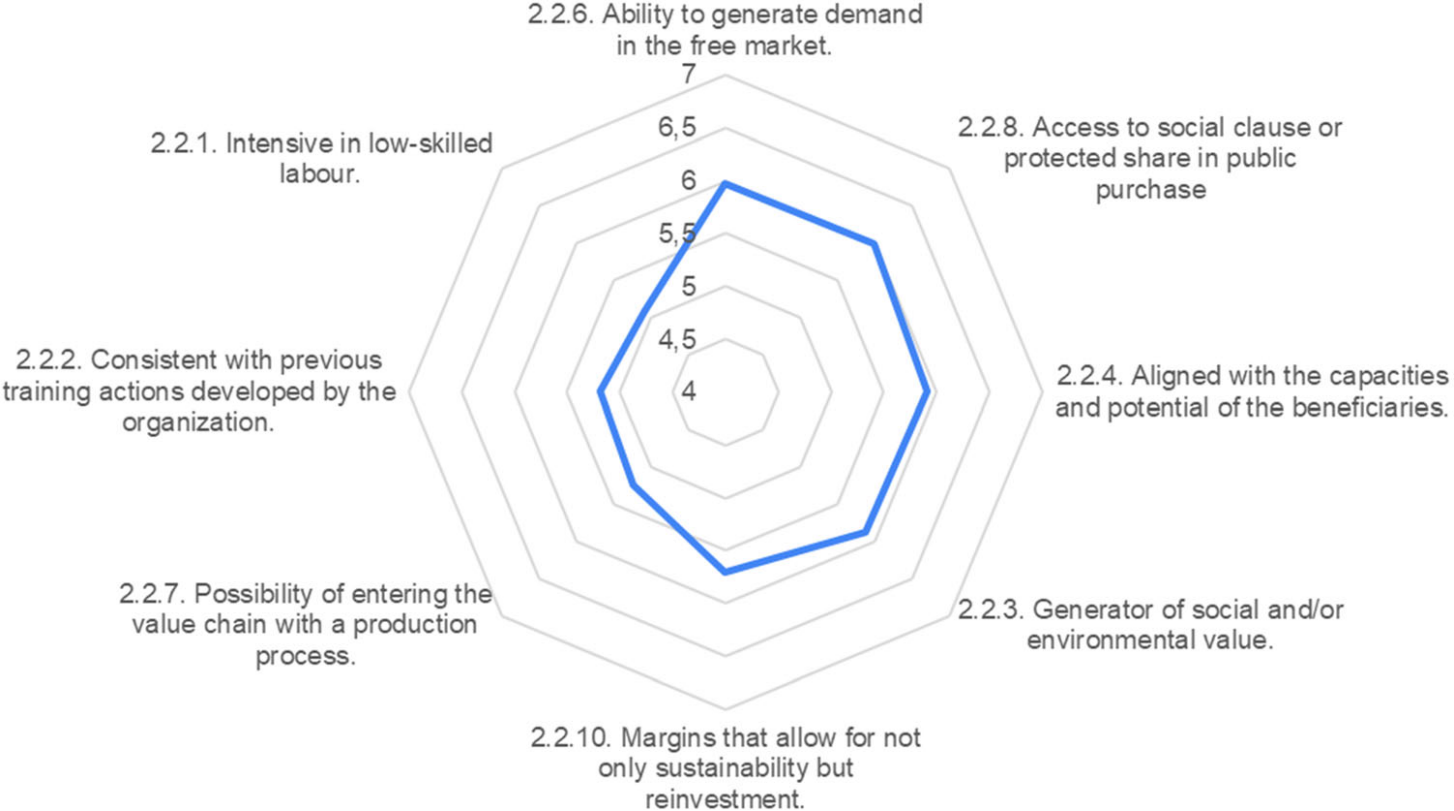
Characteristics of the economic activity

There is no consensus among the experts (var > 2) that the selected activity must necessarily fill a gap not occupied by the public sector or not profitable for the private sector, even though there is consensus that such criterion should be considered neutral when making the decision. The same happens with the proposition that the venture should need a low investment, which would tend to rule out industrial activities; in fact, more and more WISEs are carrying out industrial activities, generating more job stability (Fig. 9).

In the third phase, the criteria to be evaluated are those related to the determination to launch the company. They are more operational and involve compliance with regulations. It is no longer so much about the decision to create the company, but rather what steps to take and what options to pursue in each specific case. Here, the consensus among the experts consulted was much less, since in each promoting entity the circumstances and specific criteria diverge.

The assessments of the specific characteristics of the company to be created and the rest of the elements that facilitate the creation process are shown in “Appendix 3”. In this block, the experts were consulted on a key element for this research, as it validates the hypothesis that social entrepreneurship as an organizational process does not depend so much on the determination of one person, a specific intra-entrepreneur, but rather on a strategic decision taken within the organization’s management board or fellows’ general assembly (5.65).

There was near unanimity on the need to carry out a business plan (6.35) as a first step, which, in addition to the usual components, must in the case of social enterprise also anticipate the generating of social and environmental value (5.92), thus being useful, not only to plan the start-up strategy and calculate potentialities and risks, but also to obtain support from civil society and public administrations (5.47).

Although it did not reach consensus level, the high valuation obtained for the company’s need to share resources and processes with the promoting entity, at least in the beginning (5.55), is worth noting. In fact, it is common practice that part of the individualized social insertion itinerary that workers in a WISE follow during their time with the company is carried out in collaboration with the promoting organization.

The same is true with the question of which legal formula is used. Although there is no consensus among the experts consulted, in practice the corporation legal formulas are more relevant to WISEs than cooperative formulas (5.02). In fact, most WISEs created in Spain operate as limited liability companies (FAEDEI, 2018), perhaps due to their transitory nature that makes it difficult for most workers to acquire the status of member of a cooperative, or since a WISE must be majority-owned by its promoting entity, which leads in many cases to the adoption of the one-person limited partnership formula. However, the same does not apply in other countries such as Italy, where the cooperative formula is more frequently employed. Therefore, many experts do not perceive it as a more important criterion, regardless of what the national law dictates.

**Fig. 10:**
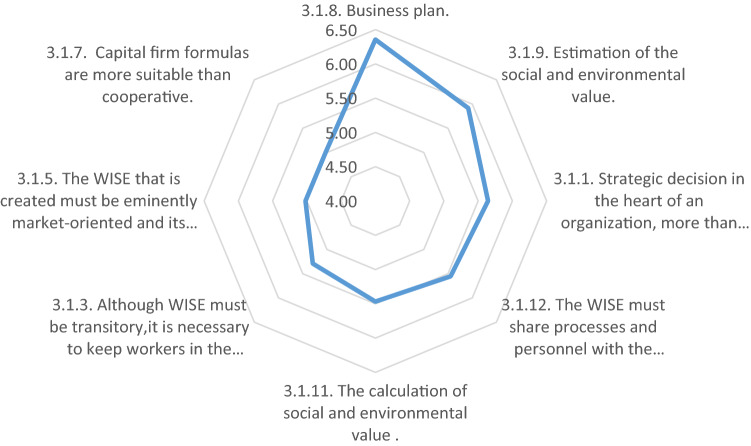
Elements that facilitate the creation of the WISE by its promoting entity

Other elements that made for theoretical debate before the passage of the Spanish law and that, once legally resolved, continue to divide experts include, for example, the transitory nature of WISEs (López-Aranguren, 1999) because, although their objective is placement in the normalized labor market, even for the most difficult cases, a company must retain some of its insertion workers to capitalize on their knowledge of the job for the benefit of the company’s competitiveness (5.29). There is also the market orientation of the companies’ activity (Ruiz Roqueñi et al., 2007), because although they must compete, and their proceeds must come mainly from economic activity (5.02), public administrations can legally support their contribution to the active employment policies through social clauses or market quotas protected in the current regime of public procurements (Fig. 10).

Finally, from a more domestic perspective, we asked the experts to what extent the requirements established in the Spanish law for WISEs, extrapolable to other European countries, make their creation difficult.

In this block, the scale changes with 1 being “makes easier” and 7 “makes more difficult” the creation of the WISE. The lack of consensus of the experts on this group of elements is noteworthy even though, in the second round, four of them modified their answers in this block alone.

In any case, and despite the lack of agreement, the valuation is very low, less than or equal to 4. Therefore, compliance with the requirements established by law can be understood as guarantors of the social companies’ nature and as an entry barrier to promoters of WISEs, with motivations closer to commercial than to social criteria.

With the reservations already noted, we can carry out an analysis of the data expressed in “Appendix 4”. Although these factors are not considered as difficulties, but rather as neutral, it should be noted that the lowest-valued elements have to do with the transitory nature of the WISE (4.46), key in its nature as a structure of the insertion itinerary—in fact, it must appear expressly in its corporate purpose (3.90)—but that these elements give rise to a very heterogeneous staff structure (4.21) and the need to have an individualized itinerary (3.52), which makes its management more complex, and even more so if the condition of the workers has to be certified and supervised by social service agencies (4.21).

Responses in a second group refer to companies’ administrative registration obligations (3.90) in each autonomous community (3.58) that requires submission of an annual report with a threefold economic, social, and environmental balance (3.36), which allows the companies to avail themselves of the subsidized WISE work contract (3.31) and have access to the aid that each region grants for their contribution to active employment policies.

**Fig. 11:**
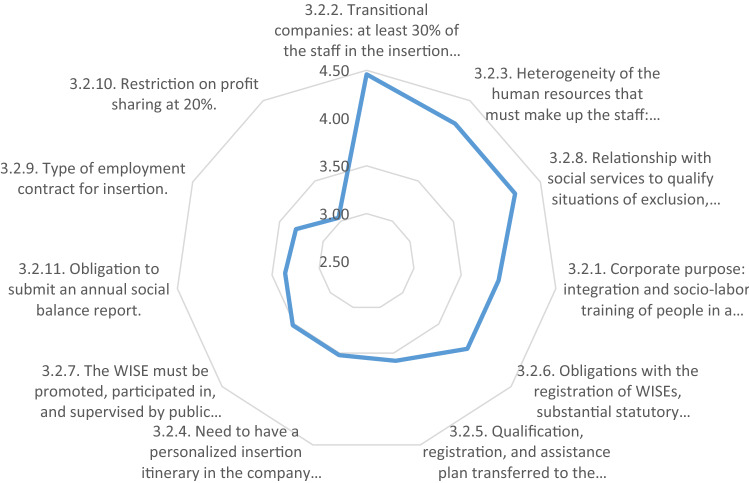
Elements that hinder the creation of the WISE by its promoting entity

Finally, it should be noted that the obligations derived from the need to guarantee the WISE’s nature as a non-profit social enterprise obtain the best evaluation. That is, it must be promoted by and have the active participation of a non-profit entity (3.52), and 80% of its profits must be reinvested in productive activity or in the improvement of its social services (3.04) (Fig. 11).

## Conclusions

The evolution of the Third Sector toward social enterprise, which started in the final decade of the twentieth century, was accelerated and conditioned by the crises of the second decade of the twenty-first century. With this, organizational social entrepreneurship rose to the fore both as an object of study and as a process to be described and transmitted to the managers of social entities that are faced with the decision to create a social enterprise, promoted by and with the participation of the organization in which they provide service.

This decision process, like other strategic processes, takes elements and criteria iteratively from the environment and from the organization itself. The social problems and civil or institutional commitment are the environmental factors that stimulate and condition the process, but the analysis of resources and capabilities of the organization will determine the final decision.

The economic activity must be subordinate to the social challenge and generator of value for both its clients and beneficiaries as well as for stakeholder groups of the promoting entity. Therefore, the link between the promoting entity and the social enterprise, which guarantees its non-profit nature and its usefulness for the social purpose, introduces participative decision-making processes that make its organization and management more complex.

The literature in social entrepreneurship has focused on individual decisions, but, in this context, it is necessary to provide new models and tools for decision makers in the Third Sector in order to stimulate the creation of social enterprises as new emerging mechanisms for solving social problems.

In this paper, we have tried to provide a three-phased model explicative of the process of organizational social entrepreneurship. The first phase is the conceptual, where the analysis of the causes of the social problem must show the creation of a social enterprise as the best possible solution although the promoter entity was not created with an entrepreneurial goal. The second phase is aimed at analyzing the concrete opportunity in terms of the future viability of the project. In this phase, it is necessary to analyze the resources from the environment and the human, methodological, and financial attributes of the organization itself. Finally, in a third phase, decisions must be made about the viability of the company to be created. In this phase, the entity must choose the optimal options in terms of legal compliance, business activity, and invested resources.

In this study, experts involved in the creation of work insertion social enterprises in Spain have validated this three-phase model and confirmed that this is a decision made by an organization and not an individual process. We conducted this study by proposing to experts the evaluation of 54 environmental, organizational, and commercial items.

The main environmental factors that determine the desirability of Spanish WISE creation are the changes in the labor market post-crisis, the general acceptance of the entrepreneurial role of the Third Sector entities, and the good results of this kind of company in labor insertion.

But also, a favourable environment provides some necessary assets for entrepreneurial activity: civil society commitment, socially responsible purchasing, investment, and philanthropy of companies or citizens. Methodological support and recognition of the phenomenon by public administrations also contribute to the perception of viability in the decision-making process.

After analyzing the external elements, it is necessary to verify organizational ones. The entrepreneurial leadership, culture, and commitment of the managers are essential to start the process. Therefore, the capacity of human resources is the key factor to continue, mainly the socio-labor situation of the beneficiaries and their ability to develop an economic activity, but also the possibility of involving technical staff in entrepreneurial activity. The innovative methodological (job training) and financial assets complete the perception that a company can be created by a non-profit social action organization.

Finally, the determination to create a new social enterprise requires choosing a specific business activity. It must be, first of all, appropriate to the skills of the beneficiaries and, if possible, consistent with previous job training activities. The promoters seek activities with secure demand, through alliances with private sector social value chains, or social public purchases, to reduce promotion efforts. They prefer activities capable of contributing social and environmental value, with margin enough for continuous reinvestment, substantial generation of employment, and ability to compete in the free market without public support.

As a formula recognized and protected by authorities in Spain, work insertion social enterprises are highly regulated by national and regional laws. The experts consulted consider this regulation necessary for the non-profit status and social purpose guarantee, and neutral in terms of difficulty for the creation of new companies.

In future research, the model needs to be validated by other non-profit organizations that have not created a social enterprise, to find out if this model improves their entrepreneurial intention and if it helps them to make the decision, or why they decide not to start the entrepreneurial process.

## Footnotes

1 The number of WISEs in Spain was determined by the authors’ own elaboration, consulting the regional registers and the information offered by the regional associations. The figure of 250 companies corresponds to those registered in July 2020. Some authors (European Commission, 2020) consider both Special Employment Centers and Labor Insertion Enterprises as WISEs, but the word ‘insertion’ makes reference to the transition to regular labor markets, while Special Employment Centers are permanent protected employment structures. In consequence, we only consider transitional structures.

2 As a synthetic example of a business model canvas for a social enterprise, see: https://www.socialbusinessmodelcanvas.com/.

3 As defined by Osterwalder and Pigneur (2010): customer segment, value proposition, distribution channels, customer relationship channels, revenue model, key activities, key resources, relationships and alliances, and cost structure.

4 The generally accepted definition of Social Enterprise in Europe is the one provided by EMES Network (Borzaga and Defourny, 2001): ‘Organizations with the express intention of benefiting the community, initiated by a group of citizens and in which the material interest of investors is subject to limits’, with four enconomic or entrepreneurial dimensions and five social dimensions. https://emes.net/focus-areas/social-enterprise/.

5 In the Spanish case, the definition and criteria of a WISE are reflected in the regulations governing such ventures: Law 44/2007, of December 13, and the respective regional regulations.
